# Improving Skin Cancer Classification Using Heavy-Tailed Student T-Distribution in Generative Adversarial Networks (TED-GAN)

**DOI:** 10.3390/diagnostics11112147

**Published:** 2021-11-19

**Authors:** Bilal Ahmad, Sun Jun, Vasile Palade, Qi You, Li Mao, Mao Zhongjie

**Affiliations:** 1 School of Artificial Intelligence and Computer Science, Jiangnan University, Wuxi 214122, China; bilalahmad@stu.jiangnan.edu.cn (B.A.); 7171905005@stu.jiangnan.edu.cn (Q.Y.); wxmaoli@jiangnan.edu.cn (L.M.); 7181905016@stu.jiangnan.edu.cn (M.Z.); 2 Centre for Computational Science and Mathematical Modelling, Coventry University, Coventry CV1 5FB, UK; ab5839@coventry.ac.uk

**Keywords:** variational autoencoder, generative adversarial networks, melanoma detection, skin cancer classification, student t-distribution, heavy-tailed distribution, t-distribution, informative noise vector, deep learning, convolutional neural networks, GANs, VAE

## Abstract

Deep learning has gained immense attention from researchers in medicine, especially in medical imaging. The main bottleneck is the unavailability of sufficiently large medical datasets required for the good performance of deep learning models. This paper proposes a new framework consisting of one variational autoencoder (VAE), two generative adversarial networks, and one auxiliary classifier to artificially generate realistic-looking skin lesion images and improve classification performance. We first train the encoder-decoder network to obtain the latent noise vector with the image manifold’s information and let the generative adversarial network sample the input from this informative noise vector in order to generate the skin lesion images. The use of informative noise allows the GAN to avoid mode collapse and creates faster convergence. To improve the diversity in the generated images, we use another GAN with an auxiliary classifier, which samples the noise vector from a heavy-tailed student t-distribution instead of a random noise Gaussian distribution. The proposed framework was named TED-GAN, with T from the t-distribution and ED from the encoder-decoder network which is part of the solution. The proposed framework could be used in a broad range of areas in medical imaging. We used it here to generate skin lesion images and have obtained an improved classification performance on the skin lesion classification task, rising from 66% average accuracy to 92.5%. The results show that TED-GAN has a better impact on the classification task because of its diverse range of generated images due to the use of a heavy-tailed t-distribution.

## 1. Introduction

Melanoma is the least common but most brutal of all skin lesions, with the highest mortality rate per year worldwide [1]. With effective early-stage diagnoses, the survival rate of patients increased substantially; reported to be 98.5% [2]. In contrast, the 5-year survival rate decreases to 19.9% only, with the failure of timely detection of melanoma [2]. Patients’ skin color and similarities among various skin lesions make it hard for medical experts to diagnose it correctly at the initial stage. Highly expert dermatologists can diagnose melanoma visually with an accuracy of 60% only [3].

A technique with better accuracy commonly used by dermatologists is dermoscopy. It is an in-vivo and non-invasive technique that eliminates skin surface reflection and magnifies it up to 400% for better examination. Dermoscopy is also called epiluminescence microscopy or dermatoscopy. In this method, the diagnosis accuracy of melanoma is up to 84% [4,5]. It is a technique with better accuracy but is time and resource-consuming, and the diagnosis efficiency is easily affected by various physical and human factors [6]. Therefore, it is necessary to develop more accurate and faster computer-aided diagnosis (CAD) systems to reduce the burden on healthcare systems and assist dermatologists in making better decisions.

In classic CAD and machine-based systems, researchers use several image processing filters to extract an image’s characteristics and features. For example, the Harris Corner detector is a Gaussian window function for detecting the edges and corners of the image. Median or averaging filters were used to reduce the noise. Such methods were difficult, time-consuming, and useful for small data sets only. In addition, it was difficult to transfer the algorithm’s learning to another unseen challenge.

Recently, Convolutional Neural Networks (CNNs) and image processing techniques gained tremendous attention from researchers of all the fields of science due to their good performance, especially in medical imaging applications [7,8,9,10,11,12]. Deep learning models are composed of many layers and can have millions of parameters. The deeper the model, the more data is needed to train it. Otherwise, the model starts to exhibit overfitting. Overfitting is a common problem in deep learning architectures that are trained on small datasets.

A CNN-based end-to-end system requires a large quantity and high quality of labeled data to function well. Having a high-quality labeled dataset, especially in medicine, is expensive and time-consuming. Melanoma, for example, is the most serious type of skin cancer. In actual practice, only 20% of patients are diagnosed with melanoma following a biopsy, and not all patients go for a biopsy in the first place [6]. This indicates that if a biopsy is not recommended to the patient in the first place, the odds of melanoma growing and progressing to the worst stage increase; conversely, recommending a biopsy for each patient increases the burden on the healthcare system. To address the limited availability of medical data and overfitting, researchers focus on testing and proposing novel ways to use deep learning models with small datasets.

Data augmentation and transfer learning are two clear strategies that academics are increasingly employing to handle the challenge of limited data. In these methods, researchers extend the training set in data augmentation by modifying the training images by scaling, cropping, and rotating them. Transfer learning involves using a pre-trained model that has already been trained on a big dataset and retrains a few layers of the model on a small training set. It works well for classification tasks involving common generic species such as cats, dogs, trees, etc. Naturally, this is not the case with a medical dataset. We do not usually have pre-trained models that can be employed for transfer learning in medical image classification tasks because of the restricted availability and difficulty of obtaining medical datasets [13,14].

One of the possible solutions recently adopted by the researchers to solve the limited availability of medical datasets is to generate artificial data. Among several generative models, Generative Adversarial Networks (GANs) [15] have gained the attention of medical image processing researchers. Recently, GANs were used in medical image (MI) generation [16,17], medical image editing in latent space [18], MI segmentation [19], and MI classification [20], because of their better performance among all generative models. A typical GAN consists of a generator (G) and a discriminator (D) network. The generator’s job is to produce more realistic images from random noise by learning the distribution of real images. In contrast, the discriminator’s job is to differentiate whether the images fed to it are real or fake (generated by the generator). They are trained alternatively to reach the final convergence. Its popularity is mainly because it automatically learns the image distribution in an unsupervised manner without employing, for example, a Markov Chain Monte Carlo (MCMC) approximation. Another beauty of GANs which makes them stand out in several traditional generative models is that they generate the image as a whole, not pixel by pixel, which provides more diversity in the generated images.

Mode collapse, on the other hand, is a common issue in GANs. They need a significant amount of training data to avoid this. In the event of mode collapse, a GAN is unable to generate clear images. Some scholars have addressed these issues and proposed GAN extensions [21]. All of them used random Gaussian noise as an input to sample the noise vector for the generator. Because random Gaussian noise does not have a strong tail, the image distribution learned from it cannot guarantee diverse image production.

Heavy-tailed distributions such as the Cauchy, log-normal, and t-distributions have been utilized to replace the random noise Gaussian distribution with excellent results. Student t-distribution is an infinite mixture of Gaussian distribution. The t-distribution was utilized in machine learning research as a replacement for the random noise Gaussian distribution [22,23]. Because of its extended tail features, it achieves excellent results in terms of diversity and low computational cost. For example, among all other income distributions, log t-distribution provides the greatest fit for predicting the income distribution of European Union countries [22]. Van et al. presented a modification to Stochastic Neighbor Embedding that uses the t-distribution to show high-dimensional data in a low-dimensional latent space by assigning a place in a two-dimensional map to each data point. This modification was much faster to optimize because of the t-distribution instead of Gaussian noise [23].

Even though numerous enhancements to GANs have been developed, the noise distribution it uses to sample the input noise vector is still random Gaussian distribution. This area, input noise distribution, has not yet been explored in generative models.

**Figure 1:**
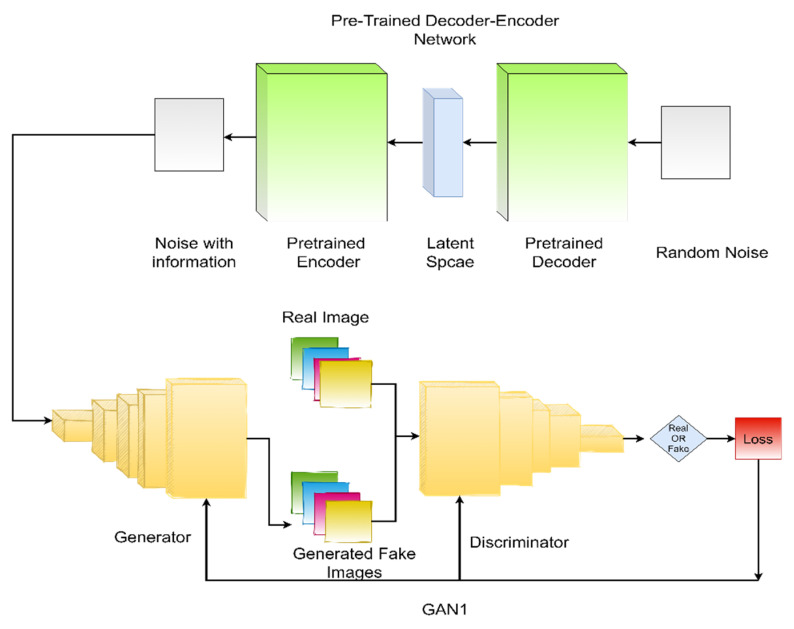
Encoder-decoder of VAE is swapped after the training so that output noise from the decoder could be used as an input to the generator of GAN_1_. The figure represents the VAE and the GAN_1_ of the proposed methodology. It’s a partial block diagram representation of the proposed TED-GAN.

**Figure 2:**
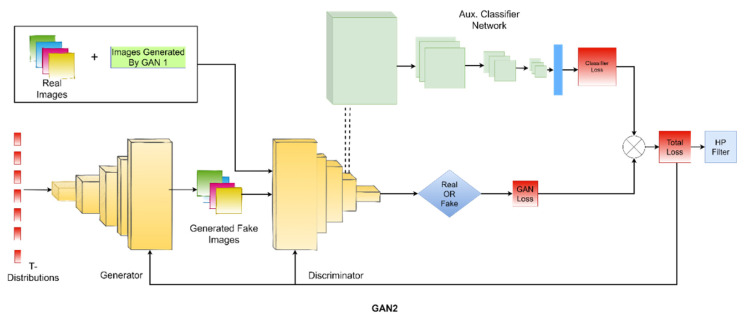
The generative adversarial network, GAN_2_, of the proposed framework and the auxiliary classifier. GAN_2_ samples the input noise vector from student t-distribution and uses the images generated by GAN_1_ for training. Generated images are passed through a high pass filter to improve imperceptibility.

We used two generative adversarial networks (GANs) and one variational autoencoder (VAE) in the proposed methodology in this paper. As VAEs are easy to train compared to GANs, we first train the variational autoencoder (VAE) separately on our training dataset. Then, we swapped the encoder-decoder network into a decoder-encoder network and let the GAN_1_ (the first generative model of our framework) sample the input from the output of VAE. This way, GAN_1_ has fewer chances to collapse and tends to adopt the domain information easily. For the training of our main generative adversarial network GAN_2_, we used student t-distribution instead of random Gaussian noise. GANs tend to perform better generally if we have a large amount of training data. We increased the training data of GAN_2_ by using the images generated by GAN_1_ at the previous stage. Furthermore, we added an auxiliary classifier network to the discriminator. The main generative adversarial network (GAN_2_) and the auxiliary classifier were trained together. The block diagram of the proposed methodology is presented in Figure 1 and Figure 2. The main contributions of this work can be summarized as follows:

- We used the VAE network to produce a noise vector that has the domain information.
- We used heavy-tailed student t-distribution to add diversity in the generated medical images.
- We used an auxiliary classifier to push the network to produce images from a specific category.
- To the best of our knowledge, this is the first time that, instead of using random noise, a separate network was trained to obtain domain information and used that informative noise for the generation of medical images.

The rest of this paper is organized as follows. Section 1.1 reviews some previous work related to generative models, including GANs and their applications in medical image generation. Section 2 reports the proposed TED-GAN and experiment settings in detail. Results, discussion, and conclusion are presented in Section 3, Section 4 and Section 5, respectively.

### 1.1. Related Work

Before the dawn of generative adversarial networks (GANs), Deep Belief Networks (DBNs), Restricted Boltzmann Machines (RBMs), Deep Boltzmann Machines (DBMs), Generative Stochastic Network (GSN), Autoencoders (AE), Denoising Autoencoders (DAEs), and variational autoencoders (VAEs) were heavily used for generating new samples of image data. Among them, several generative models are modeled by Markov Chain Monte Carlo (MCMC) based approximations. When the gradient vanishes during the training process, MCMC-based algorithms approximate the gradient of log-likelihood [24]. In image and video datasets, the likelihood-based approaches face the curse of dimensionality. Moreover, the Markov chain approximation in high dimensional spaces is inaccurate, blurry, and computationally slow [25].

In 2007, Hays et al. [26] proposed a scene completion algorithm that uses huge data of non-annotated images. The algorithm was efficient enough to find similar images from the database to complete the task of scene completion in the host image.

These traditional algorithms have a limited capacity of image estimation, as they generate the new images pixel-to-pixel instead of estimating the image as a whole.

Deep Autoencoders are based on deep learning in which the encoder converts the input images into a latent representation. It reduces the dimensionality of an input representation, and the decoder tries to reconstruct the original images from their latent representation. The objective of the autoencoders is to reduce the reconstruction error.

A variation of autoencoders was proposed in 2013 by Kingma et al. [27], named variational autoencoders (VAEs). In a variational autoencoder, the encoder maps the image representation into a Gaussian vector, and the decoder maps the noise vector to the new image. These generative models suffer from the problem of generating blurry images.

However, with the advent of deep neural networks, image generative models have been revitalized in recent years. Particularly, Generative Adversarial Networks (GANs) have shown promising results in synthesizing realistic images [15]. To improve the image generating capability of GANs, researchers proposed various variations of GANs.

In a deep convolutional generative adversarial network (DCGAN), Radford et al. integrated a convolution operation into the GAN to improve GAN’s performance [15,21]. Moreover, DeLiGAN samples the noise vector from random noise and inputs it into the generator to improve the diversity of generated images [28]. Ma et al. combined the meta-learning with CGAN and proposed a new variant of GAN called MetaCGAN. MetaCGAN can transfer the information and style it learned during training on a large dataset to the new task with a small dataset [29]. Recently, some researchers used recurrent and convolutional neural networks (RNNs, CNNs) to generate high-resolution images. However, the algorithms generate the images pixel by pixel instead of generating images as a whole [30,31].

#### GAN Applications in Medical Imaging

The GAN and GAN-based networks that generate synthetic medical images have become very popular recently, as they solve the problem of limited availability of medical datasets. Liu et al. [32] proposed a variant of Cycle GAN that uses the Pseudo Cycle consistent module and the domain control module to generate the Computed Tomography (CT) images. In this approach, the Pseudo Cycle consistent module controls the consistency of generated images, and the domain control module provides additional information of the domain.

Jiang et al. [33] proposed a Fused Attentive GAN (FA-GAN) for generating and reconstructing super-resolution magnetic resonance (MR) images. He introduced local and global feature extraction modules at different levels to extract useful features. In FA-GAN, they used 40 sets (consisting of 256 slices) of 3D magnetic resonance (MR) images for training the network, and PSNR and SSIM are used as performance measure matrices.

Ting et al. used the GAN synthesized ultrasound images of the breast and used them at the augmentation stage in the classification problem of breast lesions [20].

Ali et al. proposed Cascade Ensemble Super-Resolution CESR-GAN to reconstruct the high-resolution skin lesion images from low-resolution images. They introduced a new lossfunction based on features of the images [16].

Simulation of medical images in diverse areas of medicine is a very challenging and hot area of research these days. Synthesizing mass images in mammograms is one of them. Shen et al. used GANs to produce mass images and then fill them with contextual information by adding the synthetic lesions to healthy mammograms. They claim that their proposed network could learn the shape, context information, and distribution of real images [17]. Other researchers used GANs in various fields of medicine, including Mahapatra et al. [34], who generated retinal fundus images; Shin produced abnormal MRI images [35]; Han et al. used two-step GAN to produce MR images of brain segments with and without tumors [36]; Nie et al. generated pelvic CT images [37].

GANs have been used widely in various fields of medical imaging. Researchers try to make improvements in results by using heavy and deep architectures. Playing around the input noise of GANs is still unexplored in medical imaging.

## 2. Materials and Methods

### 2.1. Proposed Method

In this section, we introduce our proposed framework, TED-GAN, in detail. As VAEs are easy to train compared to GANs, we first train the variational autoencoder (VAE) on our training dataset to let the network store the information of the image manifold. Then we swap the network from an encoder-decoder to a decoder-encoder network. When the random Gaussian noise vector passes through this trained decoder-encoder network, it now produces a noise vector that is no longer random, but has the information of the domain. Zhong et al. [38] proved mathematically that the noise produced by swapping the encoder-decoder network has the information of image manifold. GAN_1_ would sample the noise vectors from this informative noise to produce realistic images. GAN_1_ now has a very minute chance of collapse and tends to adopt the domain information easily as its input is sampled from a latent vector of trained VAE instead of random noise.

For the training of our main generative adversarial network GAN_2_, we used student t-distribution instead of random Gaussian noise. GANs tend to perform better generally if we have a large amount of training data. So, we used the images generated by GAN_1_ along with real images of the training set (images from the HAM10000 dataset [39]) to feed the discriminator. Furthermore, we added a classifier network in front of the discriminator that shares the feature layer of the discriminator. This is called an auxiliary classifier. The main generative adversarial network (GAN_2_) and the auxiliary classifier are trained together. In the end, generated skin lesion images passed through a high pass filter to improve the imperceptibility. The small step of adding the high pass filter enhanced the quality of images significantly. The whole framework consists of one decoder-encoder network, two GANs, and one classifier. We name it TED-GAN; T from t-distribution, and ED from the encoder-decoder network.

**Figure 3:**
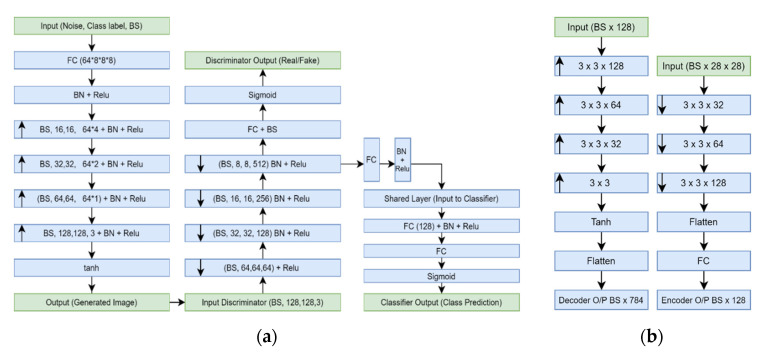
↑, ↓, BS, FC, BN, and O/P indicate upsampling, downsampling batch size, fully connected layer, batch normalization, and output, respectively. (**a**) The architecture of Generative adversarial networks used in this study. (**b**) Variational autoencoder architecture.

**Figure 4:**
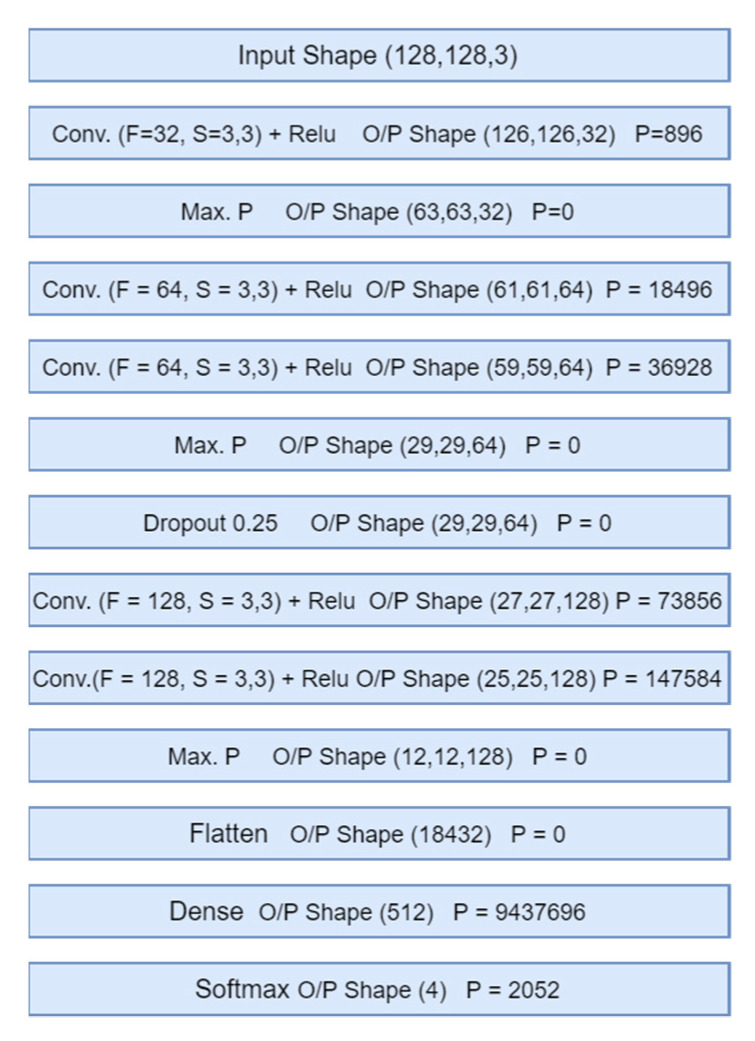
The architecture of a CNN classifier is used to test the classification performance of different generative models. Here P, Max. P, F, and S represent the parameters, maximum pooling, number of filters, and filter size, respectively.

The TED-GAN is used to generate skin lesion images only. We built a separate CNN classifier consisting of a few layers for the fair comparison of classification results with other generative models. This classifier was used to compare the performance of various generative models with the proposed one. The architecture details of the TED-GAN and the block diagram of the CNN classifier are presented in Figure 3a,b and Figure 4, respectively.

#### 2.1.1. Variational Autoencoders (VAEs)

The variational autoencoders (VAEs) consist of two parts; the encoder and decoder. The encoder consists of a separate network that samples the data 𝓍 from original data and tries to learn the latent representations (𝓏), whereas the decoder network tries to reconstruct the original image 𝓍’ from the latent representation 𝓏 [40].

(1) $$𝓏\to Enc\left(𝓍\right)=𝓆\left(𝓏|𝓍\right)\;\tilde{𝓍}\to \mathrm{Dec}\left(𝓏\right)=P\left(𝓍|𝓏\right)$$

Typically, 𝓏 is sampled from Gaussian distribution N(0,1). The VAE objective function consists of two terms, reconstruction error and a regularization term, given in the following equation.

(2) $$L_{\left(\mathrm{VAE}\right)}=-E_{𝓆(𝓏|𝓍)}[\log\frac{P\left(𝓍|𝓏\right)P\left(𝓏\right)}{𝓆(𝓏|𝓍)}]$$

(3) $$=-E_{𝓆(𝓏|𝓍)}[\log P(𝓍|𝓏)]\;+D_{kl}\left({𝓆}_{\varphi }(𝓏|𝓍)||P(𝓏)\right)$$

where the term −$E_{𝓆(𝓏|𝓍)}\log P(𝓍|𝓏)]$ is called reconstruction error (L_(Rec)_) and $D_{kl}\left({𝓆}_{\varphi }(𝓏|𝓍)\|P(𝓏)\right)$ is the Kullback-Leibler divergence.

#### 2.1.2. GAN with Student T-Distribution

As we discussed in the introduction, heavy-tailed t-distribution tends to produce better results than random Gaussian noise. In this section, we will discuss the reparameterization trick of t-distribution. In generative models, backpropagation does not hold as it is. To sample the noise from a student t-distribution instead of a standard normal distribution and to reduce the generative and discriminative loss, we propose a reparameterization of the latent generative space, using a mixture of students’ t-distributions. The probability density function (pdf) of multivariate student t-distribution is given by

(4) $$f\left(x\right)=\frac{\Gamma \left[\frac{1}{2}\left(d+n\right)\right]}{\Gamma \left(\frac{d}{2}\right)\;{\left(d\pi \right)}^{\frac{n}{2}}\;{\left|\Sigma^{*}\right|}^{\frac{1}{2}}}{\left[1+\frac{\left(\underline{x}-\underline{\mu }\right)\prime {\left(\Sigma^{*}\right)}^{-1}\left(\underline{x}-\underline{\mu }\right)}{d}\right]}^{-\frac{d+n}{2}}$$

(5) $$f\left(x\right)=\frac{\Gamma \left(\frac{d+n}{2}\right)}{\Gamma \left(\frac{d}{2}\right)\;d^{\frac{n}{2}}\;\pi^{\frac{n}{2}}\;{\left|\Sigma^{*}\right|}^{\frac{1}{2}}}{\left[1+\frac{1}{d}{\left(\underline{x}-\underline{\mu }\right)}^{\prime }{\left(\Sigma^{*}\right)}^{-1}\left(\underline{x}-\underline{\mu }\right)\right]}^{-\frac{d+n}{2}}$$

where $\underline{\mu },n,\;d\;\&{\;\Sigma }^{*}$ represents its parameters and is given by:

Mean vector $\underline{\mu }=$ ($\mu_{1},\mu_{2},\ldots ,\mu_{n}$)

Number of variables = n

Degree of freedom = d

Positive-definite symmetric matrix ${\;\Sigma }^{*}\;of\;size=n\times n$, respectively.

Covariance matrix ($\Sigma_{s}^{*}$) of variables that follow the pdf of t-distribution with a degree of freedom d>2 is given by

$\Sigma_{s}^{*}=d^{\prime }\Sigma^{*}\;where\;d^{\prime }=d{\left(d-2\right)}^{-1}\;for\;d>2$. Therefore, multivariate student t-distribution can be written as $\underline{t}\left(\underline{\mu },d\prime \Sigma^{*}\right)$ with parameters definite matrix ($\Sigma^{*}$), degree of freedom ($d^{\prime }$) and mean vector ($\underline{\mu }$).

A t-distribution $\underline{t}\left(\underline{\mu },d\prime \Sigma^{*}\right)$ is said to be a standard t-distribution if it has a mean vector equal to zero ($\underline{\mu }=\underline{0}$), covariance matrix equal to the identity matrix ($\Sigma^{*}=\underline{Ι}$), and can be written as $\underline{t}\left(\underline{0},d\prime \underline{Ι}\right)$. Where $\underline{0}$ is a vector with all zeros and $\underline{Ι}$ is an identity matrix.

To sample a noise vector, we first randomly select one of the t-distributions and then sample an n-dimensional vector from the selected t-distribution. Thus, as [41] claimed as well, sampling of noise vector and linear transformation of t-variable still follows the t-distribution. Thus, sampling a noise vector z^*^→$\underline{t}\left(\underline{\mu_{i}},d^{\prime }\Sigma_{i}^{*}\right)$ from the general t-distribution becomes equivalent to sampling ε^*^→$\underline{t}\left(\underline{0},d^{\prime }\underline{Ι}\right)$ and calculating z^*^ according to Equation (6).

(6) $$z^{*}=\underline{\mu }+\Sigma_{i}^{*}\varepsilon^{*},\;\varepsilon^{*}∊\underline{t}\left(\underline{0},d\prime \underline{Ι}\right)$$

So, the distribution $P_{z*}(z*)$ of latent noise can be written as:

(7) $$P_{z*}(z*)=\sum_{i=1}^{N}\pi_{i}\underline{t_{i}}(\underline{\mu_{i}},d\prime \Sigma_{i}^{*})$$

where *N* represents the number of t-distributions and $\pi_{i}$ is the weight of each element. We assume $\Sigma_{i}^{*}$ is a diagonal matrix and each diagonal element is initialized with non-zero value of 0.03, close to the suggestion of Sun et al. [42]. We initialize each element of $\underline{\mu_{i}}$ by sampling from a uniform distribution of range from −1 to 1. So, this way, $\underline{\mu_{i}}$ and $\Sigma_{i}^{*}$, both can be learned during the learning process of other parameters of the proposed network TED-GAN.

#### 2.1.3. The Loss Function

To push TED-GAN to produce an image from a particular category, we need to put some extra information or condition to both generator (G) and discriminator (D). We represent the output of the generator as G(z′|l) where ‘l’ represents the label of the category to push the network to produce the images from a specific class. So, the generator loss $G_{l}$ and the discriminator loss $D_{l}$ is given in Equations (8) and (9), respectively.


(8) $$G_{l}=-E_{z\prime |l∼P_{z\prime |l}}[\log(D(G(x|l)))]$$

(9) $$D_{l}=-E_{x∼P_{data}}[\log(D(x)]\;-E_{z\prime |l∼P_{z\prime |l}}[\log(1-D(G(x|l)))]$$

#### 2.1.4. Auxiliary Classifier Loss Function

As the class label information is encoded into the network, we can add an auxiliary classifier to the discriminator of TED-GAN. In this way, we can push the discriminator to do two tasks; identifying whether it is a real or fake image and predicting the class label of the image. Making the network do additional jobs proved good practice for improving network performance in the basic tasks. In our case, the auxiliary classifier does the additional supervisory job to push the generator and discriminator to produce realistic and diverse skin lesion images.

Our auxiliary classifier $L_{c}$ shares the feature extraction layers with the discriminator, and its loss function is given by [43]


(10) $$L_{c}=-E_{x∼P_{data}}[\log(D(x)]\;E_{z\prime |l∼P_{z\prime |l}}[\log D(G(z\prime |l))]$$

### 2.2. Experiment Settings

For experiments, we used an i7-6850 processor supported by the Graphical Processing Unit (GeForce Nvidia GTX 1080 GPU), with an operating system, Ubuntu 18.04.2 LTS, installed on it. We wrote the code in python programming language V3.8 with external libraries, including Keras, TensorFlow v2.0.0, Sci-kit-learn, Pandas, NumPy, and Matplotlib.

We used the HAM10000 dataset [39] for skin cancer to evaluate the proposed methodology and conducted extensive experiments. This is a benchmark dataset for skin cancer images that consists of more than 10,000 images of 7 types of skin lesions. We used the four categories, basal cell carcinoma (Bcc, 514 images), benign keratosis (Bkl, 1099 images), melanocytic nevus (Nv, 6705 images), and melanoma (Mel, 1113 images). The other three categories of lesions in this dataset do not have enough images to train a GAN. We split the dataset into training, validation, and test sets with 60%, 20%, and 20% of the data, respectively.

The training set, i.e., 60% of the dataset, is used for VAE and GAN_1_ training. After the training of the encoder-decoder network, the network is swapped into the decoder-encoder network. Now the output of the decoder consists of a noise vector, not an image. This noise vector (the output of VAE) that has the information of the image manifold is used in the training of GAN_1_. Additionally, the images generated by GAN_1_ are used in the training of GAN_2_. There is no difference in the architectures of the GAN_1_ and GAN_2_, except that the GAN_2_ has an auxiliary classifier. Moreover, GAN_2_ samples the noise vector from the student t-distribution, whereas GAN_1_ samples from the pre-trained decoder-encoder latent vector.

The CNN classifier shown in Figure 4 is trained on these images generated by TED-GAN + 60% images of the HAM10000 dataset. This classifier is trained for an equal number of epochs (1000 epochs) for various image datasets generated by other generative models, and classification results are compared. The block diagrams and architecture details of the proposed framework are shown in Figure 1, Figure 2, Figure 3 and Figure 4, respectively. We used Adam as an optimizer, 0.01 learning rate, batch size 10 with categorical cross-entropy.

To generate the images from other generative models, we used their publicly available code with default parameter settings.

For performance measures, we used precision, recall, average accuracy, and F1-score. The recall is also called sensitivity. Mathematically, sensitivity, specificity, accuracy, and F1-score can be written as:

(11) $$Sensitivity\;\left(Recall\right)=\frac{TP}{TP+FN}$$

(12) $$Specificity=\frac{TN}{TN+FP}$$

(13) $$Accuracy=\frac{TP+TN}{Total}$$

(14) $$F1\;Score=2*\left(\frac{Sensitivity*Specificity}{Sensitivity+Specificity}\right)$$

where:

*FN* = False Negatives *TP* = True Positives

*TN* = True Negatives *FP* = False Positives

## 3. Results

**Figure 5:**
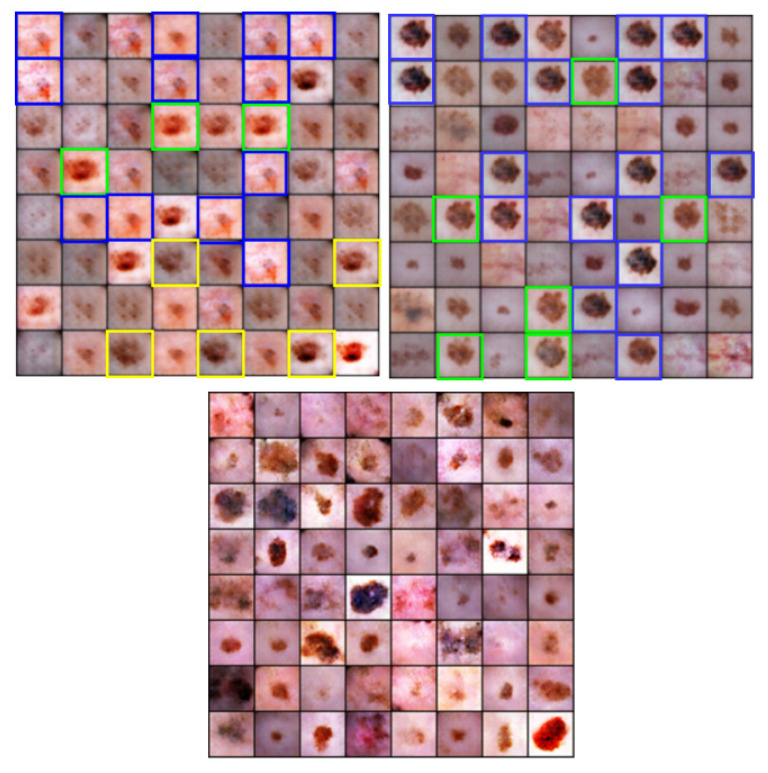
Artificially generated images of most swear type of skin cancer, melanoma by GANS. The top left, right, and bottom blocks of images represent the melanoma images generated by GAN, DeLiGAN, and TED-GAN (proposed), respectively. By having a close look at upper two blocks of images, we can observe the repetition of generated images. The green, blue, and yellow blocks contain similar images. Diversity in the images can clearly be observed visually in the proposed block (bottom one).

Generating medical images with any GAN is tricky and challenging compared to generating other images, for example, different species images, like dogs, cats, etc. Moreover, using these synthesized images to train the model for disease diagnosis makes this task more crucial. The dataset we used in this study was relatively tiny. We tried to leverage on using the t-distribution that generates diverse images because of its fatter tail. These artificially generated and diverse images played an important role in improving the results of deep learning classifiers. The artificial melanoma images generated by various generative models are shown in Figure 5. We can observe that the imperceptibility of images generated by TED-GAN is significantly better than other generative models.

Moreover, the diversity of generated images by TED-GAN (proposed) can be observed in Figure 5 (bottom row). Other generative models used in this study for comparison, GAN [15], DeLiGAN [28], generated several repetitive images highlighted in green, blue, and yellow color boxes in Figure 5. Although these repetitive images are similar in shape, they still have different textures, color temperatures, and saturation. These properties can be observed by having a close look at those highlighted images.

**Figure 6:**
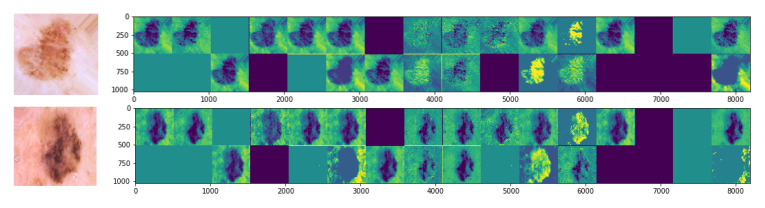
Features visualization of original and generated melanoma images. Upper and lower rows (original and generated images, respectively) represent the features that the convolutional layer of the classifier learned during the training process.

The first convolution layer of CNN learned similar features from both generated and real images. This phenomenon can be seen in Figure 6, representing the feature visualization of the real and generated image. This could be the reason that a simple GAN also has a significant impact on the classification results, though it generates several repetitive images.

A detailed quantitative comparison of average accuracy, F1-score, sensitivity, and specificity of various generative models with the proposed method are summarized in Table 1. Table 1 shows that TED-GAN performed best, followed by DeLiGAN [28] and basic GAN [15]. Some GAN variations like deep convolutional generative adversarial networks (DCGAN) [21] can generate better images, but require significantly large amounts of training data (around 60,000 images for training). So, we could not include it in our comparison.

## 4. Discussion

Melanoma is the most dangerous type of skin cancer. Dermatologists are always interested in its sensitivity and specificity. A simple CNN classifier trained on the HAM10000 images only without any generative images achieved only 53% and 75% sensitivity and specificity, respectively, for the melanoma class. In contrast, for the same class of melanoma, the sensitivity and specificity results improved to 70% and 88% (Table 1, column 4), respectively, when the images generated by GAN were included in the training of the classifier. Whereas the proposed generative model (TED-GAN) achieved 82% sensitivity and 94% specificity values of melanoma. The classification results improved around 3–5% further for all the models (GAN, DeLiGAN, and TED-GAN) when classic augmentation (cropping, scaling, and rotation) was used along with generative images for the training of the classifier.

Apart from comparing with other image generative methods, we compare our classification results with several other studies published in various journals within the last three years. The average accuracy of the proposed method is better than the others, around 2–7%. The classification results are summarized in Table 2.

**Figure 7:**
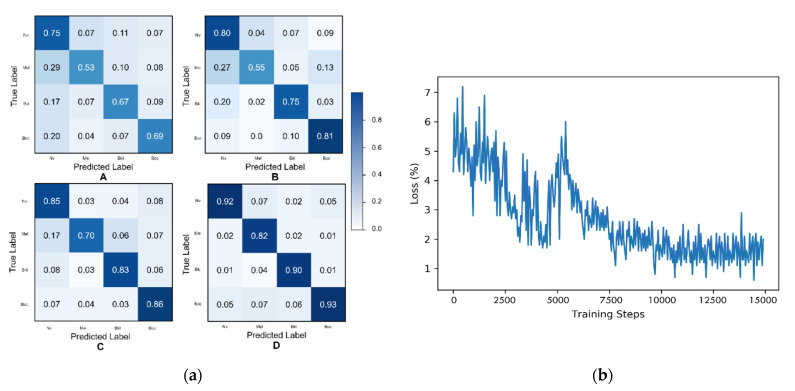
Confusion matrices of various generative models. (**a**) (**A**)—Confusion matrix of the CNN classifier when trained only on HAM10000 images without any generative image. (**B**)—When classifier training includes the HAM10000 training set and the images generated by GAN [15]. (**C**)—When classifier includes HAM10000 training set and the images generated by DeLiGAN. (**D**)— When classifier training includes the HAM10000 training set and the images generated by proposed TED-GAN. (**b**) Training loss of the generator of TED-GAN (GAN_2_).

Figure 7a represents the confusion matrices of the CNN classifier when it is trained on the images generated by various generative models, including GAn, DeLiGAN, and the proposed TED-GAN.

All the performance measures indicate that using any GAN improves the classification performance of the deep learning model. Additionally, if we use the proposed method to generate the skin lesions, results can significantly be improved because of two reasons.

First, the noise from which the TED-GAN sampled previously is already trained and contains domain information. It also reduces the training time of the TED-GAN considerably. We trained the TED-GAN only for 15,000 training steps, which is half of the time used for the training of other competing GAN models. They trained for 30,000 training steps. Figure 7b depicts the generator loss of TED-GAN. After 10,000 training steps, the average loss value stays around 1.5, which is approximately equal to 2log2, the convergence value suggested by Goodfellow [15].

Secondly, the use of the heavy tail distribution, t-distribution, gives diversity to the generated images. The diversely generated images result in better training of the CNN classifier and prevent it from overfitting on the test set.

### 4.1. Limitations

Although we tried to leverage the heavy-tailed distribution that tends to generate diverse images, these generated diverse medical images could be tricky, especially in some particular medical imaging applications like this one. There is a very minute difference in the color and texture of various categories of skin lesions. Diverse image generative models may end up generating skin lesions images that may not belong to that specific lesion category. This is a common limitation of all generative models when they deal with medical imaging.

### 4.2. Future Work

The performance of the proposed method can further be enhanced easily by using a deep architecture or by making some modifications in the architecture, for example, by using an attention mechanism or by introducing skip connection. We purposely used very simple and lightweight architecture as we had to perform extensive experimentation for a fairly large number of iterations to generate skin lesion images. With a sophisticated deep classifier network along with the proposed TED-GAN, we can further improve the classification results.

Moreover, further experiments can be performed to estimate the classifier’s generalization capacity for various unseen datasets. The results can be compared when a small portion of each dataset is included in TED-GAN training and when some of the datasets are unseen.

## 5. Conclusions

This paper proposed a framework that consists of three generative models; one VAE and two GANs. Instead of using random noise for the input of GAN, we trained the VAE to produce informative noise and let the GAN sample the input noise vector from this informative noise. This helped the adversarial network avoid mode collapse, and it converged faster. Moreover, in the second generative network, we used the heavy-tail student t-distribution. This added diversity to the generated images. The proposed framework improved the classification results of skin lesions from 66% average accuracy to 92.5% average accuracy.

TED-GAN performance was compared with other generative models and existing studies published within the last three years. It successfully achieved a better average accuracy of around 2–7% higher than other generative models.

## Figures and Tables

**Table 1:** Sensitivity, specificity, F1-score, and an average score of individual lesion class for various generative models. Nv, Mel, Bcc, and Bkl are the abbreviations of skin lesions. Please consult Section 4 for more details about the dataset and skin lesion names. Augmentation represents the classic augmentation, e.g., rotation, cropping, scaling, etc. Moreover, artificial images generated by various generative models are included in the training set.

		Nv	Mel	Bcc	Bkl	Avg. Acc
Without Augmentation						
GAN [15]	Precision	0.73	0.88	0.86	0.80	81.0%
	Recall	0.85	0.70	0.83	0.86	
	F1-Score	0.78	0.78	0.85	0.83	
DeLiGAN [28]	Precision	0.76	0.91	0.86	0.85	83.75%
	Recall	0.87	0.73	0.86	0.89	
	F1-Score	0.81	0.81	0.86	0.87	
TED-GAN (Proposed)	Precision	0.87	0.94	0.94	0.84	**89.25%**
	Recall	0.92	0.82	0.90	0.93	
	F1-Score	0.89	0.88	0.92	0.88	
With Augmentation						
GAN [15]	Precision	81	90	90	81	85.25%
	Recall	88	81	85	87	
	F1-Score	0.84	0.85	0.88	0.84	
DeLiGAN [28]	Precision	85	94	94	83	89.3%
	Recall	90	83	89	92	
	F1-Score	0.87	0.88	0.91	0.87	
TED-GAN (Proposed)	Precision	90	94	93	93	**92.5%**
	Recall	94	89	92	95	
	F1-Score	0.92	0.91	0.92	0.94	

Avg. Acc = average accuracy; Bold indicates the maximum value achieved in the respective experiment settings.

**Table 2:** Comparison of proposed TED-GAN classification results with various studies published within the last three years.

	Source	Dataset	Method	No. of Classes	Sensitivity (Recall)%	Specificity (Precision)%	F1-Score	Accuracy
2019	CNN [44]		CNN	2	92.8	68.2	-	-
	[45]	HAM10000	Physicians	5	66	62	-	-
			CNN	5	86.1	89.2	-	-
2020	[46]	Private	R-CNN	2	-	-	-	86.3
	[47]	HAM10000	GoogLeNet Inception-v3	7	75.57	-	-	-
2021	[7]	HAM10000	KELM	7	90.2	-	-	90.67
	(TED-GAN) Proposed	HAM10000	TED-GAN & CNN	4	89	94	0.91	92.5

## Data Availability

We used the pubic dataset available at doi:10.1038/sdata.2018.161.
